# *rac*-(2*R*,3*S*)-2-Phenyl-3-(3-phenyl-1,2,3,4-tetra­hydro­quinoxalin-2-yl)quinoxaline

**DOI:** 10.1107/S1600536808015481

**Published:** 2008-06-07

**Authors:** Sven Ammermann, Constantin Daniliuc, Peter G. Jones, Wolf-Walther du Mont, Hans-Hermann Johannes

**Affiliations:** a Laboratorium für Elektrooptik, Institut für Hochfrequenztechnik, Technische Universität Braunschweig, Postfach 3329, 38023 Braunschweig, Germany; b Institut für Anorganische und Analytische Chemie, Technische Universität Braunschweig, Postfach 3329, 38023 Braunschweig, Germany

## Abstract

The title compound, C_28_H_22_N_4_, is the unexpected by-product of the reaction of 2-hydroxy­acetophenone and 1,2-diamino­benzene under iodine catalysis, during which a carbon–carbon σ-bond between two quinoxaline units was formed. Although a fully oxidized title compound should sterically be possible, only one quinoxaline ring is fully oxidized while the second ring remains in the reduced form. As expected, the tetra­hydro­quinoxaline unit is not planar; it adopts a sofa conformation, whereby the atom joining the two heterocyclic systems lies out of the plane of the other atoms. The quinoxaline ring system makes a dihedral angle of 53.61 (4)° with its phenyl ring substituent. The crystal packing is determined by pairs of N—H⋯N, N—H⋯π and weak C—H⋯N hydrogen bonds, forming a chain parallel to the *a* axis.

## Related literature

For related literature, see: Banik *et al.* (1999 ▶); Chen *et al.* (2005 ▶); Gazit *et al.* (1996 ▶); Hwang *et al.* (2005 ▶); Jones *et al.* (2006 ▶); Kim *et al.* (2004 ▶); Kulkarni *et al.* (2006 ▶); McGovern *et al.* (2005 ▶); More *et al.* (2005 ▶); Raw *et al.* (2004 ▶); Robinson & Taylor (2005 ▶); Shirota & Kageyama (2007 ▶).
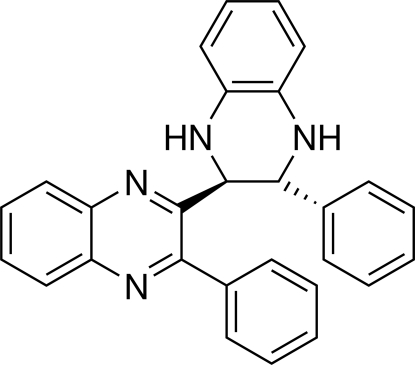


## Experimental

### 

#### Crystal data

C_28_H_22_N_4_
*M* *_r_* = 414.50
Monoclinic, 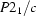

*a* = 11.1601 (6) Å
*b* = 11.3987 (6) Å
*c* = 16.4638 (8) Å
β = 93.170 (2)°
*V* = 2091.17 (19) Å^3^
*Z* = 4
Mo *K*α radiation
μ = 0.08 mm^−1^
*T* = 133 (2) K
0.35 × 0.35 × 0.32 mm

#### Data collection

Bruker SMART 1000 CCD diffractometer
Absorption correction: none
24311 measured reflections
6364 independent reflections
3306 reflections with *I* > 2σ(*I*)
*R*_int_ = 0.109

#### Refinement

*R*[*F* ^2^ > 2σ(*F* ^2^)] = 0.057
*wR*(*F* ^2^) = 0.152
*S* = 0.92
6364 reflections
297 parameters
H atoms treated by a mixture of independent and constrained refinement
Δρ_max_ = 0.42 e Å^−3^
Δρ_min_ = −0.29 e Å^−3^

### 

Data collection: *SMART* (Bruker, 1998 ▶); cell refinement: *SAINT* (Bruker, 1998 ▶); data reduction: *SAINT*; program(s) used to solve structure: *SHELXS97* (Sheldrick, 2008 ▶); program(s) used to refine structure: *SHELXL97* (Sheldrick, 2008 ▶); molecular graphics: *XP* (Siemens, 1994 ▶); software used to prepare material for publication: *SHELXL97*.

## Supplementary Material

Crystal structure: contains datablocks I, global. DOI: 10.1107/S1600536808015481/su2058sup1.cif

Structure factors: contains datablocks I. DOI: 10.1107/S1600536808015481/su2058Isup2.hkl

Additional supplementary materials:  crystallographic information; 3D view; checkCIF report

## Figures and Tables

**Table 1:** Hydrogen-bond geometry (Å, °)

*D*—H⋯*A*	*D*—H	H⋯*A*	*D*⋯*A*	*D*—H⋯*A*
N4—H02⋯N2^i^	0.84 (2)	2.49 (2)	3.211 (2)	144 (2)
C15—H15⋯N3^ii^	1.00	2.69	3.499 (2)	138
N3—H01⋯Cent(C23–C28)^ii^	0.85 (2)	2.63	3.42	157

Symmetry codes: (i) 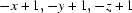; (ii) 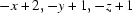.

**Fig. 1.**
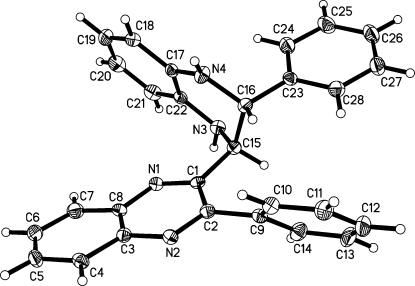
The molecular structure of compound (II), showing the atom numbering scheme and displacement ellipsoids drawn at the 50% probability level.

**Fig. 2.**
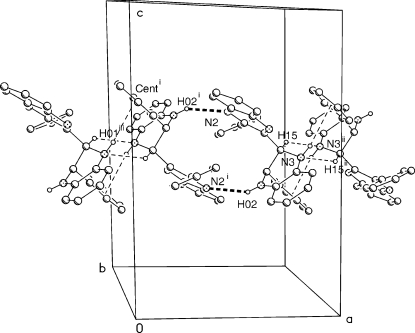
The crystal packing of compound (II), viewed approximately parallel to the b axis, showing the formation of a chain of molecules. Dashed lines indicate C—H···N, N—H···π (thin) and N—H···N (thick) hydrogen bonds. Symmetry operator (iii): -1 + x,y,z.

**Fig. 3.**
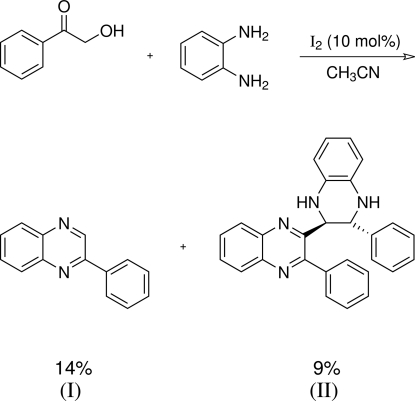
The formation of the title compound.

## supplementary crystallographic information

### Comment

Quinoxalines are a versatile class of heterocyclic compounds. This moiety is found in pharmaceutically and biologically active molecules *e.g.* as potential antibiotics (Kim *et al.*, 2004), DNA cleavage agents (More *et al.*, 2005) and for inhibition of tumor activity (Gazit *et al.*, 1996). The electron-withdrawing property of quinoxalines leads to their use in electroluminescent devices as electron transporters (Shirota & Kageyama, 2007). Often these transporters are designed as a dipolar unit consisting of an acceptor (quinoxaline) and a donor (*e.g.* triarylamines) (Chen *et al.*, 2005; Kulkarni *et al.*, 2006). Lately quinoxalines have been used as ligands for metal complexes (Jones *et al.*, 2006) that show efficient electroluminescence (Hwang *et al.*, 2005) in organic light-emitting diodes (OLEDs).

Several routes for the synthesis of quinoxalines are described in the literature. For condensations, starting materials are 1,2-diketones and 1,2-diamines which are reacted in boiling ethanol (Gazit *et al.*, 1996) or at room temperature in acetonitrile with iodine as catalyst (More *et al.*, 2005). For 2-substituted quinoxalines, α-hydroxy ketones and 1,2-diamines are used together with different catalysts such as Pd(OAc)_2_/Et_3_N (Robinson & Taylor, 2005) or MnO_2_ (Raw *et al.*, 2004). These catalysts are necessary to oxidize the alcohol from the *a*-hydroxy ketones. In the present study a synthesis for 2-phenylquinoxaline was planned under similiar conditions to those used by More *et al.* (2005) from 2-hydroxyacetophenone, 1,2-diaminobenzene and iodine without the use of an oxidization catalyst. As expected the yield of the reaction was low, but beside the anticipated product (I) the title compound (II) was formed. To the best of our knowledge neither the structure itself nor this type of formation have been described in the literature. Raw *et al.* (2004) describe the formation of an azobenzene derivative as the by-product. The most striking feature is the formation of a carbon-carbon σ-bond [C1—C15 1.536 (2) Å] between two quinoxaline moieties. Assuming that compound (I) is formed in the first place, either the attack on the C═ N bond or the reaction of (I) as a nucleophile with the starting materials could lead to the formation of a dimer. Subsequent reduction with 2 equivalents of hydrogen would form the title compound (II). Barik *et al.* (1999) demonstrated that dimeric structures starting from imines can be formed *via* a samarium-induced iodine-catalyzed reduction. The authors postulate a one electron transfer mechanism across the C═ N bond resulting in two carbon radicals merging in a pinacol type reaction. Even though these conditions cannot be found in our case, it is this reference that is most relevant to the formation of a dimer. The red color of compound (II) is remarkable and the origin is unclear, because the UV/VIS-spectrum shows no significant absorption above a maximum of 320 nm (ε = 9300 in CH_3_CN). In comparison McGovern *et al.* (2005) have shown that an intramolecular charge-transfer causes the red color of 9,14-dihydrodipyridophenazine, which possesses a moiety like the 1',2',3',4'-tetrahydroquinoxaline in the present study. The title compound potentially exists as two different diastereomers, but one of them is formed exclusively, as shown by spectroscopic evidence. We surmise that the other diastereomer is suppressed for steric reasons.

The molecular structure of compound (II) is illustrated in Fig. 1. Bond lengths and angles in the two phenyl rings and in the quinoxaline unit are normal. As expected the tetrahydroquinoxaline unit is not planar; it adopts a sofa conformation, whereby the atom joining the two heterocyclic systems lies out of the plane of the other atoms. Atoms C15 and C16 show *sp*^3 ^hybridization (angles ranged from 107.9° to 113.9°). The relative configurations at atoms C15 and C16 are *S*,*R*. The bond length C15—C16 [1.549 (2) Å] indicates a C—C single bond, whereas bond C1—C2 [1.444 (2) Å] shows the aromatic character of an oxidized ring. The phenyl rings subtend interplanar angles of 53.61 (4)° with the quinoxaline ring system [C1, N1, C8, C3, N2 and C2], and 79.01 (4)° with the tetrahydroquinoxaline ring [C16, N4, C17, C22 and N3]  (atom C15 lies 0.655 (2) Å out of this plane).

In the crystal structure of (II) the packing of the molecules is determined by weak N4—H02···N2  and C15—H15···N3  hydrogen bonds (Fig 2 and Table 1). Pairs of alternating C—H···N and N—H···N hydrogen bonds are formed across inversion centres. Additionally, there is an N—H···π contact from N3—H01 to the centroid of the phenyl ring [C23–C28]. The overall effect is to form a chain parallel to the *a* axis.

### Experimental

A 100 ml round-bottomed flask was charged with 2-hydroxyacetophenone (3.00 g, 22.034 mmol), 1,2-diaminobenzene (2.86 g, 26.441 mmol), iodine (559 mg, 2.203 mmol), and acetonitrile (30 ml). The reaction was stirred for 23h at room temperature, monitored by thin-layer chromatography and finally concentrated to dryness under reduced pressure. The dark crude product obtained was then subjected to flash column chromatography using silica gel (eluent: 6:1 *n*-hexane–EtOAc). 630 mg (14%) of 2-phenylquinoxaline, the expected product, and 390 mg (9%) of the unexpected title compound, (II), were obtained. Red crystals of (II) grew overnight from the eluted fractions of the flash column chromatography.

### Refinement

Amide H atoms were freely refined [N—H = 0.84 (1), 0.85 (1) Å]. The other H-atoms were included in calculated positions and refined using a riding model: C—H = 0.95 - 1.0 Å with *U*_iso_(H) = 1.2*U*_eq_(C).

### Crystal data

**Table:**

C_28_H_22_N_4_	*F*_000_ = 872
*M**_r_* = 414.50	*D*_x_ = 1.317 Mg m^−^^3^
Monoclinic, *P*2_1_/*c*	Mo *K*α radiation λ = 0.71073 Å
Hall symbol: -P 2ybc	Cell parameters from 3799 reflections
*a* = 11.1601 (6) Å	θ = 2–30º
*b* = 11.3987 (6) Å	µ = 0.08 mm^−^^1^
*c* = 16.4638 (8) Å	*T* = 133 (2) K
β = 93.170 (2)º	Prism, red
*V* = 2091.17 (19) Å^3^	0.35 × 0.35 × 0.32 mm
*Z* = 4	

### Data collection

**Table:**

Bruker SMART 1000 CCD diffractometer	6364 independent reflections
Radiation source: fine-focus sealed tube	3306 reflections with *I* > 2σ(*I*)
Monochromator: graphite	*R*_int_ = 0.109
Detector resolution: 8.192 pixels mm^-1^	θ_max_ = 30.5º
*T* = 133(2) K	θ_min_ = 1.8º
ω and φ scans	*h* = −15→15
Absorption correction: none	*k* = −16→16
24311 measured reflections	*l* = −23→23

### Refinement

**Table:**

Refinement on *F*^2^	Secondary atom site location: difference Fourier map
Least-squares matrix: full	Hydrogen site location: inferred from neighbouring sites
*R*[*F*^2^ > 2σ(*F*^2^)] = 0.057	H atoms treated by a mixture of independent and constrained refinement
*wR*(*F*^2^) = 0.152	*w* = 1/[σ^2^(*F*_o_^2^) + (0.0734*P*)^2^]
*S* = 0.92	(Δ/σ)_max_ = 0.001
6364 reflections	Δρ_max_ = 0.42 e Å^−^^3^
297 parameters	Δρ_min_ = −0.29 e Å^−^^3^
Primary atom site location: structure-invariant direct methods	Extinction correction: none

### Special details

**Table:**

Geometry. All e.s.d.'s (except the e.s.d. in the dihedral angle between two l.s. planes) are estimated using the full covariance matrix. The cell e.s.d.'s are taken into account individually in the estimation of e.s.d.'s in distances, angles and torsion angles; correlations between e.s.d.'s in cell parameters are only used when they are defined by crystal symmetry. An approximate (isotropic) treatment of cell e.s.d.'s is used for estimating e.s.d.'s involving l.s. planes.Least-squares planes (*x*,*y*,*z* in crystal coordinates) and deviations from them (* indicates atom used to define plane)7.0105 (0.0062) *x* + 1.5050 (0.0091) *y* - 13.1768 (0.0076) *z* = 0.4647 (0.0095)* -0.0196 (0.0009) N3 * 0.0131 (0.0012) C22 * 0.0135 (0.0011) C17 * -0.0327 (0.0012) N4 * 0.0257 (0.0009) C16 - 0.6550 (0.0023) C15Rms deviation of fitted atoms = 0.02227.1369 (0.0068) *x* - 0.2689 (0.0093) *y* + 12.0496 (0.0089) *z* = 10.3944 (0.0068)Angle to previous plane (with approximate e.s.d.) = 79.01 (0.04)* 0.0027 (0.0013) C23 * -0.0049 (0.0014) C24 * 0.0020 (0.0015) C25 * 0.0031 (0.0014) C26 * -0.0053 (0.0013) C27 * 0.0023 (0.0013) C28Rms deviation of fitted atoms = 0.00365.4792 (0.0064) *x* + 2.1540 (0.0079) *y* + 13.5331 (0.0066) *z* = 12.6941 (0.0020)Angle to previous plane (with approximate e.s.d.) = 15.63 (0.09)* -0.0411 (0.0011) C1 * 0.0366 (0.0011) C2 * 0.0019 (0.0011) N2 * -0.0370 (0.0012) C3 * 0.0330 (0.0012) C8 * 0.0067 (0.0011) N1Rms deviation of fitted atoms = 0.0304- 4.5755 (0.0081) *x* + 2.1574 (0.0088) *y* + 15.0404 (0.0054) *z* = 7.1104 (0.0101)Angle to previous plane (with approximate e.s.d.) = 53.61 (0.04)* -0.0098 (0.0013) C9 * 0.0103 (0.0013) C10 * -0.0010 (0.0014) C11 * -0.0088 (0.0014) C12 * 0.0092 (0.0014) C13 * 0.0001 (0.0013) C14Rms deviation of fitted atoms = 0.0078
Refinement. Refinement of *F*^2^ against ALL reflections. The weighted *R*-factor *wR* and goodness of fit *S* are based on *F*^2^, conventional *R*-factors *R* are based on *F*, with *F* set to zero for negative *F*^2^. The threshold expression of *F*^2^ > σ(*F*^2^) is used only for calculating *R*-factors(gt) *etc*. and is not relevant to the choice of reflections for refinement. *R*-factors based on *F*^2^ are statistically about twice as large as those based on *F*, and *R*- factors based on ALL data will be even larger.

### Fractional atomic coordinates and isotropic or equivalent isotropic displacement parameters (Å^2^)

**Table:**

	*x*	*y*	*z*	*U*_iso_*/*U*_eq_	
N1	0.73023 (13)	0.32241 (13)	0.59153 (9)	0.0174 (3)	
N2	0.55476 (13)	0.48400 (13)	0.63650 (9)	0.0168 (3)	
N3	0.90846 (13)	0.36907 (13)	0.49171 (10)	0.0173 (3)	
H01	0.9504 (18)	0.3484 (17)	0.5339 (12)	0.019 (5)*	
N4	0.70665 (15)	0.39968 (13)	0.38883 (10)	0.0187 (4)	
H02	0.639 (2)	0.408 (2)	0.3641 (15)	0.043 (7)*	
C1	0.73376 (15)	0.43303 (15)	0.56896 (10)	0.0147 (4)	
C2	0.64804 (15)	0.51743 (15)	0.59598 (10)	0.0158 (4)	
C3	0.54617 (16)	0.36728 (15)	0.65568 (10)	0.0157 (4)	
C4	0.44601 (16)	0.32623 (17)	0.69629 (10)	0.0191 (4)	
H4	0.3847	0.3792	0.7103	0.023*	
C5	0.43791 (18)	0.20925 (17)	0.71539 (11)	0.0228 (4)	
H5	0.3694	0.1811	0.7411	0.027*	
C6	0.53032 (18)	0.13035 (17)	0.69726 (11)	0.0239 (4)	
H6	0.5238	0.0498	0.7113	0.029*	
C7	0.62894 (18)	0.16901 (17)	0.65979 (11)	0.0225 (4)	
H7	0.6919	0.1160	0.6493	0.027*	
C8	0.63732 (17)	0.28797 (16)	0.63657 (10)	0.0173 (4)	
C9	0.66016 (16)	0.64540 (15)	0.58036 (11)	0.0174 (4)	
C10	0.56469 (17)	0.70799 (16)	0.54368 (11)	0.0205 (4)	
H10	0.4918	0.6686	0.5286	0.025*	
C11	0.57501 (19)	0.82717 (17)	0.52897 (12)	0.0270 (5)	
H11	0.5099	0.8690	0.5030	0.032*	
C12	0.6806 (2)	0.88530 (18)	0.55223 (13)	0.0305 (5)	
H12	0.6881	0.9668	0.5416	0.037*	
C13	0.77457 (19)	0.82530 (17)	0.59063 (13)	0.0280 (5)	
H13	0.8459	0.8659	0.6077	0.034*	
C14	0.76546 (17)	0.70532 (17)	0.60446 (12)	0.0229 (4)	
H14	0.8309	0.6640	0.6303	0.027*	
C15	0.82767 (15)	0.46513 (15)	0.50791 (11)	0.0161 (4)	
H15	0.8761	0.5330	0.5299	0.019*	
C16	0.76314 (16)	0.50187 (15)	0.42612 (11)	0.0170 (4)	
H16	0.6983	0.5586	0.4385	0.020*	
C17	0.75484 (15)	0.28781 (15)	0.39818 (11)	0.0154 (4)	
C18	0.70330 (17)	0.19060 (16)	0.35817 (11)	0.0204 (4)	
H18	0.6338	0.2006	0.3229	0.025*	
C19	0.75227 (18)	0.07975 (17)	0.36928 (12)	0.0254 (5)	
H19	0.7159	0.0143	0.3420	0.030*	
C20	0.85415 (18)	0.06402 (17)	0.42005 (12)	0.0262 (5)	
H20	0.8884	−0.0118	0.4272	0.031*	
C21	0.90571 (17)	0.15974 (17)	0.46026 (11)	0.0226 (4)	
H21	0.9756	0.1487	0.4950	0.027*	
C22	0.85742 (15)	0.27129 (15)	0.45090 (10)	0.0157 (4)	
C23	0.84954 (16)	0.56456 (16)	0.37228 (10)	0.0166 (4)	
C24	0.92716 (18)	0.50358 (18)	0.32431 (12)	0.0261 (5)	
H24	0.9247	0.4203	0.3231	0.031*	
C25	1.00828 (19)	0.56274 (18)	0.27815 (12)	0.0300 (5)	
H25	1.0612	0.5195	0.2462	0.036*	
C26	1.01289 (18)	0.68356 (18)	0.27821 (12)	0.0279 (5)	
H26	1.0687	0.7236	0.2466	0.033*	
C27	0.93552 (18)	0.74584 (18)	0.32474 (12)	0.0263 (5)	
H27	0.9373	0.8292	0.3246	0.032*	
C28	0.85472 (17)	0.68660 (16)	0.37190 (11)	0.0207 (4)	
H28	0.8026	0.7302	0.4042	0.025*	

### Atomic displacement parameters (Å^2^)

**Table:**

	*U*^11^	*U*^22^	*U*^33^	*U*^12^	*U*^13^	*U*^23^
N1	0.0193 (8)	0.0180 (8)	0.0151 (8)	−0.0017 (6)	0.0013 (6)	−0.0004 (6)
N2	0.0183 (8)	0.0170 (8)	0.0151 (7)	−0.0004 (6)	0.0007 (6)	0.0009 (6)
N3	0.0133 (8)	0.0209 (8)	0.0176 (8)	0.0021 (6)	−0.0016 (6)	0.0006 (7)
N4	0.0156 (8)	0.0164 (8)	0.0234 (9)	0.0010 (6)	−0.0053 (7)	0.0001 (6)
C1	0.0146 (9)	0.0171 (9)	0.0122 (8)	0.0002 (7)	−0.0019 (7)	−0.0019 (7)
C2	0.0153 (9)	0.0177 (9)	0.0141 (9)	−0.0007 (7)	−0.0024 (7)	0.0001 (7)
C3	0.0164 (9)	0.0160 (9)	0.0144 (9)	−0.0005 (7)	−0.0017 (7)	0.0007 (7)
C4	0.0176 (9)	0.0245 (10)	0.0151 (9)	−0.0013 (8)	0.0009 (7)	0.0018 (8)
C5	0.0251 (11)	0.0251 (11)	0.0182 (10)	−0.0085 (8)	0.0008 (8)	0.0049 (8)
C6	0.0350 (11)	0.0190 (10)	0.0177 (10)	−0.0048 (9)	0.0002 (8)	0.0033 (8)
C7	0.0275 (11)	0.0199 (10)	0.0200 (10)	0.0014 (8)	0.0013 (8)	0.0005 (8)
C8	0.0211 (9)	0.0192 (9)	0.0115 (8)	−0.0020 (7)	−0.0001 (7)	0.0008 (7)
C9	0.0198 (9)	0.0179 (9)	0.0150 (9)	−0.0004 (7)	0.0047 (7)	−0.0003 (7)
C10	0.0222 (10)	0.0198 (10)	0.0196 (9)	−0.0014 (8)	0.0016 (8)	−0.0004 (8)
C11	0.0371 (12)	0.0182 (10)	0.0260 (11)	0.0032 (9)	0.0039 (9)	0.0009 (8)
C12	0.0445 (13)	0.0159 (10)	0.0322 (12)	−0.0033 (9)	0.0117 (10)	−0.0005 (9)
C13	0.0296 (11)	0.0207 (11)	0.0345 (12)	−0.0098 (9)	0.0089 (9)	−0.0085 (9)
C14	0.0192 (10)	0.0218 (10)	0.0277 (11)	−0.0009 (8)	0.0015 (8)	−0.0040 (8)
C15	0.0147 (9)	0.0168 (9)	0.0170 (9)	0.0002 (7)	0.0016 (7)	−0.0012 (7)
C16	0.0163 (9)	0.0169 (9)	0.0178 (9)	−0.0006 (7)	0.0010 (7)	−0.0025 (7)
C17	0.0157 (9)	0.0155 (9)	0.0152 (9)	−0.0007 (7)	0.0041 (7)	0.0009 (7)
C18	0.0230 (10)	0.0226 (10)	0.0158 (9)	−0.0028 (8)	0.0019 (8)	−0.0007 (8)
C19	0.0339 (12)	0.0174 (10)	0.0253 (11)	−0.0042 (9)	0.0057 (9)	−0.0035 (8)
C20	0.0335 (12)	0.0201 (10)	0.0258 (11)	0.0075 (9)	0.0081 (9)	0.0021 (8)
C21	0.0206 (10)	0.0252 (11)	0.0223 (10)	0.0079 (8)	0.0036 (8)	0.0021 (8)
C22	0.0141 (9)	0.0183 (9)	0.0151 (9)	0.0000 (7)	0.0040 (7)	0.0004 (7)
C23	0.0178 (9)	0.0180 (9)	0.0139 (9)	−0.0001 (7)	−0.0004 (7)	0.0019 (7)
C24	0.0328 (12)	0.0214 (10)	0.0249 (11)	−0.0028 (9)	0.0091 (9)	−0.0006 (8)
C25	0.0378 (13)	0.0285 (12)	0.0250 (11)	0.0005 (10)	0.0137 (9)	0.0007 (9)
C26	0.0279 (11)	0.0299 (12)	0.0264 (11)	−0.0059 (9)	0.0059 (9)	0.0075 (9)
C27	0.0315 (12)	0.0212 (11)	0.0259 (11)	−0.0033 (9)	−0.0010 (9)	0.0054 (8)
C28	0.0225 (10)	0.0197 (10)	0.0200 (9)	0.0026 (8)	0.0006 (8)	0.0007 (8)

### Geometric parameters (Å, °)

**Table:**

N1—C1	1.316 (2)	C21—C22	1.386 (3)
N1—C8	1.365 (2)	C23—C24	1.390 (3)
N2—C2	1.323 (2)	C23—C28	1.392 (3)
N2—C3	1.372 (2)	C24—C25	1.389 (3)
N3—C22	1.406 (2)	C25—C26	1.378 (3)
N3—C15	1.453 (2)	C26—C27	1.382 (3)
N4—C17	1.389 (2)	C27—C28	1.396 (3)
N4—C16	1.445 (2)	N3—H01	0.85 (2)
C1—C2	1.444 (2)	N4—H02	0.84 (2)
C1—C15	1.536 (2)	C4—H4	0.9500
C2—C9	1.489 (2)	C5—H5	0.9500
C3—C8	1.409 (2)	C6—H6	0.9500
C3—C4	1.414 (2)	C7—H7	0.9500
C4—C5	1.374 (3)	C10—H10	0.9500
C5—C6	1.413 (3)	C11—H11	0.9500
C6—C7	1.364 (3)	C12—H12	0.9500
C7—C8	1.413 (3)	C13—H13	0.9500
C9—C10	1.392 (3)	C14—H14	0.9500
C9—C14	1.398 (3)	C15—H15	1.0000
C10—C11	1.386 (3)	C16—H16	1.0000
C11—C12	1.387 (3)	C18—H18	0.9500
C12—C13	1.376 (3)	C19—H19	0.9500
C13—C14	1.391 (3)	C20—H20	0.9500
C15—C16	1.549 (2)	C21—H21	0.9500
C16—C23	1.523 (2)	C24—H24	0.9500
C17—C18	1.397 (3)	C25—H25	0.9500
C17—C22	1.411 (2)	C26—H26	0.9500
C18—C19	1.385 (3)	C27—H27	0.9500
C19—C20	1.385 (3)	C28—H28	0.9500
C20—C21	1.385 (3)		
			
C1—N1—C8	117.67 (15)	C25—C26—C27	119.30 (19)
C2—N2—C3	117.53 (15)	C26—C27—C28	120.16 (18)
C22—N3—C15	116.50 (15)	C23—C28—C27	120.93 (18)
C17—N4—C16	122.39 (15)	C22—N3—H01	111.2 (13)
N1—C1—C2	121.38 (16)	C15—N3—H01	112.2 (13)
N1—C1—C15	116.44 (15)	C17—N4—H02	119.4 (16)
C2—C1—C15	122.04 (15)	C16—N4—H02	118.0 (16)
N2—C2—C1	121.10 (16)	C5—C4—H4	120.2
N2—C2—C9	116.80 (15)	C3—C4—H4	120.2
C1—C2—C9	122.10 (16)	C4—C5—H5	119.6
N2—C3—C8	120.67 (16)	C6—C5—H5	119.6
N2—C3—C4	119.80 (16)	C7—C6—H6	119.8
C8—C3—C4	119.52 (16)	C5—C6—H6	119.8
C5—C4—C3	119.55 (17)	C6—C7—H7	120.0
C4—C5—C6	120.76 (17)	C8—C7—H7	120.0
C7—C6—C5	120.43 (18)	C11—C10—H10	119.7
C6—C7—C8	119.95 (18)	C9—C10—H10	119.7
N1—C8—C3	121.07 (16)	C10—C11—H11	120.1
N1—C8—C7	119.10 (17)	C12—C11—H11	120.1
C3—C8—C7	119.71 (17)	C13—C12—H12	119.9
C10—C9—C14	118.95 (17)	C11—C12—H12	119.9
C10—C9—C2	120.16 (16)	C12—C13—H13	119.9
C14—C9—C2	120.86 (16)	C14—C13—H13	119.9
C11—C10—C9	120.61 (18)	C13—C14—H14	119.9
C10—C11—C12	119.83 (19)	C9—C14—H14	119.9
C13—C12—C11	120.24 (19)	N3—C15—H15	108.8
C12—C13—C14	120.20 (19)	C1—C15—H15	108.8
C13—C14—C9	120.13 (18)	C16—C15—H15	108.8
N3—C15—C1	113.18 (14)	N4—C16—H16	107.7
N3—C15—C16	107.91 (14)	C23—C16—H16	107.7
C1—C15—C16	109.39 (14)	C15—C16—H16	107.7
N4—C16—C23	113.96 (15)	C19—C18—H18	119.6
N4—C16—C15	108.80 (14)	C17—C18—H18	119.6
C23—C16—C15	110.72 (14)	C18—C19—H19	119.9
N4—C17—C18	121.95 (16)	C20—C19—H19	119.9
N4—C17—C22	119.13 (16)	C21—C20—H20	120.3
C18—C17—C22	118.91 (16)	C19—C20—H20	120.3
C19—C18—C17	120.83 (18)	C20—C21—H21	119.3
C18—C19—C20	120.17 (18)	C22—C21—H21	119.3
C21—C20—C19	119.48 (18)	C25—C24—H24	119.5
C20—C21—C22	121.39 (18)	C23—C24—H24	119.5
C21—C22—N3	121.97 (17)	C26—C25—H25	119.7
C21—C22—C17	119.21 (17)	C24—C25—H25	119.7
N3—C22—C17	118.81 (16)	C25—C26—H26	120.3
C24—C23—C28	118.01 (17)	C27—C26—H26	120.3
C24—C23—C16	122.03 (16)	C26—C27—H27	119.9
C28—C23—C16	119.94 (16)	C28—C27—H27	119.9
C25—C24—C23	120.91 (18)	C23—C28—H28	119.5
C26—C25—C24	120.69 (19)	C27—C28—H28	119.5
			
C8—N1—C1—C2	4.7 (2)	N1—C1—C15—N3	−6.8 (2)
C8—N1—C1—C15	−171.04 (15)	C2—C1—C15—N3	177.41 (15)
C3—N2—C2—C1	3.5 (2)	N1—C1—C15—C16	113.51 (17)
C3—N2—C2—C9	−176.16 (15)	C2—C1—C15—C16	−62.2 (2)
N1—C1—C2—N2	−8.0 (3)	C17—N4—C16—C23	91.8 (2)
C15—C1—C2—N2	167.59 (16)	C17—N4—C16—C15	−32.3 (2)
N1—C1—C2—C9	171.65 (16)	N3—C15—C16—N4	54.42 (18)
C15—C1—C2—C9	−12.8 (2)	C1—C15—C16—N4	−69.11 (18)
C2—N2—C3—C8	3.4 (2)	N3—C15—C16—C23	−71.57 (18)
C2—N2—C3—C4	−177.68 (16)	C1—C15—C16—C23	164.91 (14)
N2—C3—C4—C5	−179.96 (16)	C16—N4—C17—C18	−176.29 (16)
C8—C3—C4—C5	−1.1 (3)	C16—N4—C17—C22	5.0 (3)
C3—C4—C5—C6	2.1 (3)	N4—C17—C18—C19	−179.23 (17)
C4—C5—C6—C7	−0.5 (3)	C22—C17—C18—C19	−0.6 (3)
C5—C6—C7—C8	−2.0 (3)	C17—C18—C19—C20	−0.5 (3)
C1—N1—C8—C3	2.2 (2)	C18—C19—C20—C21	0.7 (3)
C1—N1—C8—C7	178.17 (16)	C19—C20—C21—C22	0.1 (3)
N2—C3—C8—N1	−6.6 (3)	C20—C21—C22—N3	179.64 (17)
C4—C3—C8—N1	174.52 (16)	C20—C21—C22—C17	−1.1 (3)
N2—C3—C8—C7	177.48 (16)	C15—N3—C22—C21	−152.96 (17)
C4—C3—C8—C7	−1.4 (3)	C15—N3—C22—C17	27.8 (2)
C6—C7—C8—N1	−173.07 (17)	N4—C17—C22—C21	−179.94 (16)
C6—C7—C8—C3	2.9 (3)	C18—C17—C22—C21	1.3 (3)
N2—C2—C9—C10	−53.0 (2)	N4—C17—C22—N3	−0.7 (2)
C1—C2—C9—C10	127.41 (18)	C18—C17—C22—N3	−179.40 (16)
N2—C2—C9—C14	124.95 (19)	N4—C16—C23—C24	−40.5 (2)
C1—C2—C9—C14	−54.7 (2)	C15—C16—C23—C24	82.5 (2)
C14—C9—C10—C11	2.0 (3)	N4—C16—C23—C28	141.30 (17)
C2—C9—C10—C11	179.92 (17)	C15—C16—C23—C28	−95.7 (2)
C9—C10—C11—C12	−1.1 (3)	C28—C23—C24—C25	0.7 (3)
C10—C11—C12—C13	−0.7 (3)	C16—C23—C24—C25	−177.50 (18)
C11—C12—C13—C14	1.7 (3)	C23—C24—C25—C26	−0.7 (3)
C12—C13—C14—C9	−0.8 (3)	C24—C25—C26—C27	−0.1 (3)
C10—C9—C14—C13	−1.0 (3)	C25—C26—C27—C28	0.8 (3)
C2—C9—C14—C13	−178.92 (17)	C24—C23—C28—C27	0.0 (3)
C22—N3—C15—C1	66.7 (2)	C16—C23—C28—C27	178.24 (16)
C22—N3—C15—C16	−54.46 (19)	C26—C27—C28—C23	−0.8 (3)

### Hydrogen-bond geometry (Å, °)

**Table:**

*D*—H···*A*	*D*—H	H···*A*	*D*···*A*	*D*—H···*A*
N4—H02···N2^i^	0.84 (2)	2.49 (2)	3.211 (2)	144 (2)
C15—H15···N3^ii^	1.00	2.69	3.499 (2)	138
N3—H01···Cent(C23–C28)^ii^	0.85 (2)	2.63	3.42	157

Symmetry codes: (i) −*x*+1, −*y*+1, −*z*+1; (ii) −*x*+2, −*y*+1, −*z*+1.
